# Prevalence of depression and associated factors among epileptic patients at Ilu Ababore zone hospitals, South West Ethiopia, 2017: a cross‑sectional study

**DOI:** 10.1186/s12991-020-00268-5

**Published:** 2020-03-11

**Authors:** Nigus Alemnew Engidaw, Lemi Bacha, Adamu Kenea

**Affiliations:** 1 College of Medicine and Health Sciences, Debre Berhan University, Debre Berhan, Ethiopia; 2 Department of Psychiatry, Faculty of Public Health and Medical Science, Mettu University, Mettu, Ethiopia

**Keywords:** Prevalence, Depression, Epilepsy, Ethiopia

## Abstract

**Background:**

Depression is one of the most common and overwhelming mental disorder in patients with epilepsy. Despite its high prevalence, depression continues to be under-recognized and undertreated. This study aimed to assess the prevalence of depression and its associated factors among epileptic patients attending the outpatient department of Ilu Ababore zone hospitals, Southwest Ethiopia, 2017.

**Methods:**

Institution-based cross-sectional study was carried out among 402 individual with epilepsy. The participants were selected using systematic random sampling technique. Depression was measured using Beck’s Depression Inventory II. Oslo 3 Social Support Scale was used to assess social support. Perceived Stress Scale was used to assess the stress level of epileptic patients. The data were entered into Epi Info version 7 and analyzed by the SPSS version 20 software. We computed bivariate and multivariate binary logistic regressions to assess factors associated with depression. Statistical significance was declared at *p*-value < 0.05.

**Results:**

A total of 402 study participants were interviewed with a response rate of 96.2%. The prevalence of depression was found to be 48.1%. In the final multivariate analysis, educational status [unable to read and write (AOR = 4.01,95% CI = 3.82, 8.28), primary (AOR = 3.43, 95% CI = 3.12,9.29), secondary (AOR = 2.01, 95% CI = 1.89,7.24)], high perceived stress (AOR = 3.21, 95% CI = 2.70, 8.41), poor social support (AOR = 2.04, 95% CI = 1.42, 2.78), onset of illness < 6 year (AOR = 2.40, 95%CI = 2.10,7.91), seizure frequency of [1–11 per year (AOR = 2.34, 95% = 1.41, 4.36), ≥ 12/year (AOR = 3.49, 95% CI = 3.43, 6.40)], and polytherapy (AOR = 2.73, 95%CI = 2.52, 7.14) were independent predictors of depression among epileptic patients at *p*-value < 0.05.

**Conclusion and recommendation:**

Overall, the prevalence of depression was found to be high. Having lower educational status, early onset of illness, poor social support, high perceived stress, high seizure frequency, and polytherapy were factors statistically associated with depression. Clinicians need to give emphasis to epileptic patients with high perceived stress, low educational status, and poor social support. An early depression-focused regular screening for epileptic patient should be carried out by trained health professionals. Linkage with mental health service providers also needs to be considered.

## Background

Epilepsy is one of the most known neurological disorders which is characterized by recurrent seizures [1]. It can be related with undesirable physical, social, and psychological consequences. Epilepsy also has an influence on an individual's quality of life [2]. The presence of recurrent seizures may cause difficulties in important areas of the patient’s life and hindering the development of new relationships. More than 70 % of people with epilepsy become seizure-free with treatment of antiepileptic medications [3–5]. The prevalence of epilepsy in Africa is estimated between 2.2 and 58/1000 population and constitutes the second or third reason for consultation and hospitalization [6]. In Ethiopia, it occurs in about 5.2/1000 population [7, 8].

There is a high incidence of psychiatric comorbidity in people with epilepsy (PWE), particularly depression [9]. Among the 50 Mio. PWE worldwide, 9.5% to 85% are also likely to suffer from depressive disorders. Out of this, more than 80% of them reside in low-income regions where psychiatric comorbidities are often under-recognized and undertreated [10–21]. Depression is the leading cause of years lived with disability and the fourth leading cause of disability-adjusted life-years worldwide [22]. It is more often seen in epilepsy than in the general population [23–25]. People with epilepsy experience depression at two to three times the rate of the general population [26]. Depression is characterized by loss of interest, depressed mood, disturbance of sleep, problem in appetite and psychomotor activity, difficulty to concentrate or make decision, excessive guilty or sinful feeling, easily tiredness and recurring thoughts of death or suicide [27].

Despite its high prevalence and impact, depression continues to be under-recognized and undertreated. There are no clearly established mechanisms by which epilepsy may lead to clinical depression. It has been suggested that structural abnormalities in different brain parts, monoamine pathways, cerebral glucose metabolism, the hypothalamic–pituitary–adrenal axis, and interleukin-1b are associated with the pathogenesis of depression in PWE [28–31].

The presence of depression among PWE can be associated with different psychosocial difficulties on patients’ life such as poor treatment adherence, poor quality of life, unemployment, lower educational status, increased burden and cost on healthcare services and higher risk for suicide [32–34]. Even though many studies from the Western world have reported the prevalence of depression among PWE and its negative consequences, few studies have addressed the issue in sub-Saharan countries.

In Ethiopia, the authors could find only two previously published studies concerning the prevalence of depression and its associated factors among epileptic patients that reported from the northwest and central part of Ethiopia [13, 14]. Yet, in the south western part of the country where the culture of the community is fairly different from the northwestern and central part of Ethiopia, there is a need to understand the prevalence and its contributing factors of depression. The result of this study would help to design more effective programs in the management and prevention of comorbid epilepsy and depression. Those who might be aimed to inquire the relationship between depression and epilepsy also benefited from this study. Thus, the main purpose of this study was to assess the prevalence and associated factors of depression among people with epilepsy in Ilu Ababore zone hospitals (Mettu Karl referral and Darimu hospital), south west Ethiopia, 2017.

## Methods

### Study design, period and area

An institution-based cross-sectional study design was carried out. The study was conducted from June 1 to August 30, 2017 in Ilu Ababore zone hospitals (Mettu Karl referral and Darimu general hospitals). These hospitals are located in the southwest part of Ethiopia. Mettu, the zone city, is located 600 km far from the capital city of Ethiopia, Addis Ababa. The hospital has also given the psychiatric service at the outpatient level.

### Participants

All PWE who were on follow-up at the outpatient department of Ilu Ababore zone hospitals (Mettu Karl referral and Darimu hospital) were invited to take part in the interview. Clients with a confirmed clinical diagnosis of epilepsy and age above or equal to 18 years, having outpatient regular follow-up visit at the two hospitals were considered as eligible candidates for participation. Those who could not communicate because of the illness were excluded from this study.

### Study variables

The dependent variable was depression. Independent variables included socio-demographic factors (age, sex, marital status, ethnicity, religion, residence, educational and occupational status), clinical factors (age at onset of illness, duration of the illness, treatment duration, seizure frequency, type of antiepileptic treatments, number of antiepileptic treatment and family history of mental illness), and psychosocial factors (social support and perceived stress).

### Sample size determination and sampling procedures

Single population proportion formula was used to estimate the minimum numbers of samples required for this study. The sample size was calculated by using 45.2% prevalence of depression among peoples with epilepsy attending psychiatric clinic of Gondar university teaching hospital, north west Ethiopia [14], 0.452 P, 1.96 Z (standard normal distribution), 95% CI, ⍺ = 0.05, and a 10% non-response rate. Accordingly, a representative sample was calculated to be 418.

### Sampling technique and procedure

Participants were selected for interviews using the systematic random sampling technique. Before the data collection, the total number of epileptic patients who visited the hospital in 2016 was identified from patients’ record. The average flow of epileptic patients in 2 months period at the two hospitals during the data collection period was estimated to be 872 (592 from Mettu Karl Referral hospital and 281 from Darimu Hospital).

Based on this, a sampling interval (K) was determined by diving the total number of individuals with epilepsy expected to have a follow-up visit during 8 weeks data collection period to the calculated sample size (K = 872/418≈2). The first case was selected by lottery method from the first and second patients. Then eligible individuals were interviewed for every 2 intervals based on the order of their clinical evaluation at the outpatient department until the required sample size was reaching.

### Operational definitions

Those who scored greater than or equal to 10 on BDI-II were considered as having depression [35]. Poor, moderate and high social support was considered for participants who scored 3–8, 9–11 and 12–14, respectively, out of 14 based on Oslo 3 Social Support Scale [36]. High perceived stress was considered for those who scored greater than 20 on PSS [8].

### Data collection instruments and procedures

Data were collected by face-to-face interview using a pre-tested semi-structured questionnaire consisting socio-demographic factors, clinical characteristics, Oslo 3 item social support scale and Perceived Stress Scale questioners. The outcome variable (depression) was evaluated by Beck's Depression Inventory (BDI-II). BDI-II is one of the most recent and widely used self-report measures of depression. It is a reliable and valid measure of depression in a range of cultural groups and has been validated with both psychiatric and non-psychiatric populations in most of the countries including Africa. BDI-II used for screening of recent (during past 2 weeks) depressive symptoms correspond to DSM-IV criteria in persons with epilepsy. The tool consists of 21 items, and each of its items describes a specific symptom of depression. Each statement is scored on a 4-point scale (0–3) and a total score is obtained by summing the ratings for each statement [35]. Depression was defined using a cutoff point ≥ 10 on BDI-II as having depression. A score from 0 to 9 is considered to be within the normal range or asymptomatic; a score of 10–18 indicates mild depression; a score of 19–29 indicates moderate depression and a score of 30 or more severe depression [37]. Even though BDII is not validated in the Ethiopia population, it has been used in previous similar research which was done on epileptic patient in Northwest Ethiopia. It had an internal consistence of Cronbach’s alpha 0.856 for the total score [14]. In the current study, the Cronbach’s alpha for this particular tool was 0.812.

The social support level of study participants was assessed by using the Oslo 3 Social Support Scale (OSSS-3). The tool has three item questions collectively used to measure the accessibility of support patients will receive from their family, friends, and neighbors if needed [36]. Even though Oslo 3 Social Support Scale is not formally validated in Ethiopian context, it has been used in different clinical and community-based studies of African countries including Ethiopia. It has a total sum score ranging from 3–14 points. Based on the total sum score, the level of social support was categorized as poor, moderate and high social support with sum scores of 3–8, 9–11 and 12–14, respectively.

Perceived Stress Scale (PSS) was used to assess the stress level of PWE. It is the most widely used tool for screening stress. The items in the PSS asked about the feelings and thoughts of the patients during the past month. Each item is rated on a 5-point scale ranging from never (0) to almost always [8]. Positively worded items are reverse scored, and the ratings are summed, with higher scores indicating more perceived stress. The cutoff value for the stress limit was set ≥ 20. PSS had an internal consistence of Cronbach’s alpha for the total score of PSS = 0.793 [38]. Even though it is not directly on epileptic patients, PSS is validated among Ethiopian university students [39].

Data were collected by using pre-tested questionnaires using face-to-face interview. Four BSc psychiatry nurses for data collectors and two MSc in mental health for supervision were recruited. The questionnaires were translated into Afaan Oromo (local language) by an Afaan Oromo speaking linguist and back translation to English was performed by mental health specialist into English. The Afaan Oromo version of the questionnaires was pre-tested on 5% of patients to make it easier for the participants to understand and complete.

### Data quality management

To ensure data quality, 2 days training was given for data collectors and supervisors about the purpose of the research, how to approach study participants and how to use the questionnaire. The collected data were checked out for the completeness, accuracy, and clarity by the principal investigator and supervisors. This quality checking was done daily after data collection and correction was made before the next data collection measure. Data cleanup and cross-checking was done before analysis.

### Data processing and analysis

All collected data were checked for completeness and consistency and entered into Epi Info version 7 and then exported to SPSS version 20 software for analysis. Descriptive statistics (frequencies, tables, percentages, and means) were computed to explain the socio-demographic characteristics, clinical variables, and depression in PWE. Bivariate and multivariate logistic regression analyses were done. Variables that have *p*-value < 0. 20 in the bivariate model were entered into the multivariate analysis to avoid potential confounders. In the multivariate model, variables with *p*-values of less than 0.05 were considered as statistical predictors of depression. The odds ratio with a 95% confidence interval was used to measure the strength of the association.

## Results

### Socio-demographic characteristics of the respondents

Among a total of 418 individuals invited to participate in this study, 402 participants completed the interview properly with the response rate of 96.2%. Among the respondents, the majority 162 (40.3%) were in age range of 18–24 years, about 253 (62.9%) were male, and 323 (80.34%) were Oromo by ethnicity. Nearly two-thirds of the participants (61.9%) were married and 190 (47.3%) were attended primary school education, 153 (38.1%) were orthodox religion followers. Regarding the psychosocial characteristics, more than sixty-two percent (62.7%) of the study participants had high social support and more than half (52.2%) of them had high perceived stress (Table 1).

**Table 1:** Distribution of participants by their socio-demographic and psychosocial characteristics at Ilu Ababore zone hospitals, southwest Ethiopia, 2017 (*n* = 402)

Variables	Frequency	Percent
Age		
18–24	162	40.3
25–34	121	30.1
35–44	82	20.3
≥ 45	37	9.2
Gender		
Male	253	62.9
Female	149	37.1
Religion		
Orthodox	153	38.1
Muslim	115	28.6
Protestant	105	26.1
Others^a^	29	7.2
Marital status		
Married	246	61.2
Single	117	29.1
Divorced/widowed	39	9.7
Ethnicity		
Oromo	323	80.34
Amhara	52	12.93
Tigre	14	3.48
Gurage	9	2.23
Other^b^	4	0.99
Educational status		
Unable to read and write	114	28.4
Primary	195	47.3
Secondary	58	14.4
College and above	35	8.7
Residency		
Rural	197	49.0
Urban	205	51.0
Type of occupation		
Government employ	26	6.5
Private enterprise	234	58.2
Student	60	15.0
Unemployed	82	20.4
Social support		
High	252	62.7
Moderate	98	24.4
Poor	52	12.9
Perceived stress		
Low	192	47.8
High	210	52.2

^a^Waqefetaa, Catholic, Adventist

^b^Kefa, Wolayta

### Clinical characteristics of the respondents

More than half (54.7%) of the participants had treatment duration of less than 6 years and majority, 288 (71.6%) of the respondents had seizure frequency 1–11/year. More than one-third (35.6%) of the study subjects had the illness duration of 2 to 5 years. Majority (76.4%) of the patients were on a single antiepileptic drugs and ninety-four percent (94.52%) of the study participants were taking phenobarbital. More than ninety-five percent (95.3%) of them had no family history of mental disorders (Table 2).

**Table 2:** Distribution of participants by their clinical characteristics at Ilu Ababore zone hospitals, southwest Ethiopia, 2017 (*n* = 402)

Variables	Frequency	Percent
Age at onset of illness		
< 6 years	20	4.7
6-11 years	48	11.9
12–17 years	108	28.7
18–24 years	113	28.1
25–34 years	70	17.4
≥ 35 years	43	10.7
Duration of illness		
< 1 years	41	10.2
2-5 years	143	35.6
6–10 years	119	29.6
≥ 11	99	24.6
Treatment duration		
≤ 6 years	220	54.7
> 6 years	182	45.3
Seizure frequency per year		
0	80	19.9
1–11 per year	288	71.6
≥ 12 per year	34	8.5
Type of antiepileptic drugs		
Phenobarbital	380	94.52
Phenytoin	6	1.49
Carbamazepine	9	2.23
Sodium valproate	7	1.74
Number of antiepileptic drugs		
Monotherapy	307	76.4
Polytherapy	95	23.6
Family history of mental illness		
Yes	19	4.7
No	383	95.3

### Prevalence of depression among PWE

The prevalence of depression among diabetic patients was found to 48.1%. From 402 participants; (29.8%) were classified as mild, (17.4%) as moderate and (0.9%) were severely depressed. All of the participants were undiagnosed and not treated before for depression.

### Factors associated with depression among PWE

In this study, age, educational statuses, social support, perceived stress, the onset of the illness, seizure frequency, poly-pharmacy, and family history of mental illness were variables fulfilled the minimum requirement (≤ 0.2 significance level) for further multivariate logistic analysis. In the multivariate analysis; educational status [unable to read and write (AOR = 4.01,95% CI = 3.82, 8.28), primary (AOR = 3.43, 95% CI = 3.12,9.29), secondary (AOR = 2.01, 95%CI = 1.89,7.24)], perceived stress (AOR = 3.21, 95% CI = 2.70, 8.41), poor social support (AOR = 2.04, 95% CI = 1.42, 2.78) onset of illness < 6 year (AOR = 2.40, 95%CI = 2.10,7.91), seizure frequency of [1–11 per year (AOR = 2.34, 95% = 1.41, 4.36), ≥ 12/year (AOR = 3.49, 95% CI = 3.43, 6.40)], and polytherapy (AOR = 2.73, 95%CI = 2.52, 7.14) were factors statistically significant with depression among PWE at p value < 0.05 (Table 3).

**Table 3:** Bivariate and multivariate analysis of depression and explanatory variables among people with epilepsy at Ilu Ababore hospitals, Mettu, Ethiopia, 2017 (*n* = 402)

Variables	Depression		COR 95%CI	AOR 95%CI
	Yes	No		
Age				
18–24	69	93	0.59 (0.204, 1.35)	0.34 (0.53, 1.21)
25–34	53	68	0.62 (0.318, 1.60)	0.37 (0.24, 1.35)
35–44	54	38	1.13 (0.62, 2.26)	1.32 (0.80, 7.60)
≥ 45	15	12	1.00	1.00
Educational status				
Unable to read and write	56	58	4.67 (2.23,14.56	4.01 (3.82, 8.28)**
Primary	88	107	3.98 (2.07,12.80)	3.43 (3.12,9.29)**
Secondary	30	28	5.18 (3.05,13.90)	2.01 (1.89,7.24)*
College and above	6	29	1.00	1.00
Social support				
High	105	144	1.00	
Moderate	42	51	1.12 (0..70,1.83)	1.13 (0.33,1.22)
Poor	36	25	1.97 (0.64,3.12)	2.04 (1.42, 2.78)*
Perceived stress				
Low	53	135	1.00	1.00
High	132	82	4.10 (2.79,6.48)	3.21 (2.70, 8.41)**
Age at onset of the disease in year				
< 6 years	15	5	1.30 (0.625, 9.996)	2.40 (2.10,7.91)**
6-11 years	28	20	0.61 (0.244, 1.341)	0.95 (0.287, 3.169)
12–17 years	36	72	0.22 (0.115, 0.501)	0.55 (0.213, 1.413)
18–24 years	47	66	0.31 (0.165, 0.705)	0.85 (0.343, 2.153)
25–34 years	30	40	0.33 (0.182, 0.848)	0.84 (0.330, 2.153)
≥ 35 years	30	13	1.00	1.00
Seizure frequency per year				
0	20	60	1.00	1.00
1–11 per year	145	143	3.10 (1.776, 5.280)	2.34 (1.41, 4.36)*
≥ 12 per year	17	17	3.00 (1.257, 7.157)	3.49 (3.43, 6.40)**
Number of drug				
Monotherapy	132	175	1.00	1.00
Polytherapy	50	45	1.47 (1.484, 19.389)	2.73 (2.52, 7.14)**
Family history of mental illness				
Yes	10	9	1.86 (0.476, 3.151)	1.27 (0.30,2.18)
No	143	240	1.00	1.00

* *p* < 0.05

** *p* < 0.01

Hosmer and Lemeshow test = 0.730

## Discussion

Institution-based cross-sectional study was conducted to assess the prevalence and factors associated with depression among patients epilepsy at Ilu Ababore zone hospitals using BDII. Depression was identified in 36.02% of male patients and in 60.02% of female gender. In addition to the effect of seizure, gender-based biological, psychological and social factors might be responsible for the higher prevalence of depression among epileptic women than men.

In this study, the prevalence of depression among PWE was found to be 48.1%. Out of these, (29.8%) were classified as mild, (17.4%) as moderate and (0.9%) were severely depressed. This result is in line with the study done in Iraq (51.6%) [10], Poland (49.2%) [16], Nigeria (45%) [40] and Gondar, Ethiopia (45.2%) [14].

The finding of the current study is lower than the study done in Gaza (63%) [19], Korea (62%) [20], Pakistan (60%) [18], and Nigeria (85%) [21]. The discrepancy might be due to using different diagnostic criteria in detecting depression, different in sample size and choosing epileptic patients with different seizure types, variable frequency, and severity. For example, ICD-10 was used to assess depression in the study conducted in Pakistan and a small sample was used as compared to our study which may have overrated the prevalence of depression. In the study of Gaza, majority of the study subjects had uncontrolled type seizure resulting in high prevalence of depression.

On the contrary, the result of this study (48.1%) is higher than the study done in USA (9.5%) [10], Thailand (38.5%) [11], Egypt (25.5%) [12], Bosnia (34%) [41], Mexico (42.7%) [15] and Addis Ababa, Ethiopia (32.5%) [13]. The possible reasons for the difference may be due to use of different screening tools, cutoff points, study areas and cultures of the study participants. For instance, in the study conducted in Egypt, from the total study participants, 100 of them were health individuals taken for comparison which may lower the prevalence of depression. In USA, Hospital Anxiety and Depression scale was used to evaluate depression which has different cutoff point. The difference in study area, health care system, infrastructure, and economic development might also contribute to the lower prevalence of depression.

The second objective of this study was to identify the associated factors of depression among PWE. Accordingly, those patients who cannot read and write had more than four times (AOR = 4.01, 95% CI = 3.82, 8.28**)** odds of depression as compared to those patients who had educational status of college and above. Those patients who had educational status of primary (AOR = 3.43, 95% CI = 3.12,9.29**)** and secondary (AOR = 2.01, 95%CI = 1.89,7.24**)** had more than three and two times odds of depression as compared to those patients who had educational status of college and above, respectively. These findings were consistent with the previous studies [17, 41]. This is due to the fact that those patients with lower educational status may have poor insight about their illness and stress coping mechanisms to their illness.

The odds of developing depression among epileptic patients who had poor social support (AOR = 2.04, 95% CI = 1.42, 2.78**)** were 2.04 times more likely when compared with clients who had high social support. This might be due to the fact that social isolation reduces social support, which can have undesirable influence on physical and mental well-being including depression.

Participants with high perceived stress had more than three times (AOR = 3.28, 95%CI = 2.70, 8.41**)** odds of depression as compared to those patients who had low perceived stress. This may be due to the fact individual with high perceived stress may have lesser and inappropriate psychological adjustment when they face different stressful life situations such as unemployment, significant loss and newly diagnosed with severe illness. Many studies have also showed that stress may lead to neurotransmitters imbalance such as serotonin in the brain, which leads to the occurrence of depression [42].

Study subjects who had disease onset of less than 6 years had more than 2 times (AOR = 2.40, 95 CI = 2.10, 7.91**)** odds of depression than who had disease onset at age of 35 years and above. The findings were consistent with the studies carried out in Gaza [19]. As literatures indicated, epilepsy leads to a substantial burden on the patients’ family, the community, and society at large. These burdens manifested in many domains of their life such as physical health [43], psychosocial well-being [7, 44] and monetary problem [16, 21]. In our study, the study participants may not have enough stress coping mechanisms to the above problems. This is because, at early stage of life they may not have good knowledge and experience that help them to cope up with different cultural belief, stigma, and illness that contributed to comorbid psychiatric illnesses.

PWE who had seizure frequency of 12 and above were more than three times (AOR = 3.49, 95% = 3.43, 6.40) were more likely to develop depression as compared to those patients who had no seizure frequency. The odds of developing depression for those who had seizure frequency one and above per annual were more than two times (AOR = 2.34, 95% = 1.41, 4.36) than those patients who had no seizure frequency. These findings were in line with the previous studies conducted in different countries [12, 15, 40, 42]. The likely reason may be the presentation of epilepsy is overt, sudden and not easy to realize. This difficulty of realizing where and when the seizure come may be related with socially improper presentations including loss of bladder control, foaming from the mouth and tongue biting. These signs may lead the epileptic patent to embarrassment, depression, anxiety and other social and psychological problems.

Epileptic patients who took poly-pharmacy for their seizure treatment were more than two times (AOR = 2.73, 95% CI = 2.52, 7.14) odds of depression as compared with their counter parts. These findings were in line with studies in the past [12, 41]. The possible reason might be due to the burden and adverse effect of AEDs. In the current study, there was no association between AEDs. Based on literatures, barbiturates groups especially phenobarbital has been associated with increased risk of depression. In our study, the most prescribed drug was phenobarbital (94.52%). Studies have shown that uncontrolled seizures are associated with a greater frequency of depression than seizure-free patients. As different literatures revealed, around 60% of epileptic patients develop depression and depression also escalate risk of epilepsy up to 3- to 7-fold. Studies also showed that the relapse rate of seizures after early termination of AEDs [45]. This suggested the importance of AEDs for a proper time duration.

### Limitation of the study

This study was conducted in health facilities; hence the findings might not adequately reflect the depression of the entire epileptic patients in the community. The cross-sectional nature of the study design may not confirm a definitive cause-and-effect relationship. We did not do a detailed validation study for Beck’s Depression Inventory (BDI-II) scale. This might under or overestimate our study findings. Recall bias may be another limitation for this study.

## Conclusion

In this study, the prevalence of depression was found to be high. Having lower educational status, early onset of illness, poor social support, seizure frequency, using poly-pharmacy and having high perceived stress were factors associated with depression in PWE. Regular screening for epileptic patients should be carried out by trained health professionals and strengthening the social support and educational status of PWE should be considered.

## Data Availability

The datasets generated and/or analyzed during the current study are not publicly available due to some privacy reasons, but part of the raw datasets will be available in the recommended publicly available data repository of BMC or from the corresponding author on reasonable request.

## Abbreviations

AOR: Adjusted odd ratios
AEDs: Antiepileptic drugs
BDI-II: Beck's Depression Inventory version two
CI: Confidence interval
COR: Crude odd ratios
PSS: Perceived Stress Scale
PWE: People with epilepsy
USA: United States of America
WHO: World Health Organization
